# In the modern era of percutaneous coronary intervention: Is cardiac rehabilitation engagement purely a patient or a service level decision?

**DOI:** 10.1177/2047487317717064

**Published:** 2017-06-21

**Authors:** Abdulrahman Al Quait, Patrick Doherty, Nils Gutacker, Joseph Mills

**Affiliations:** 1 Department of Health Sciences, Faculty of Science, University of York, UK; 2 King Fahad Medical City, Riyadh, Saudi Arabia; 3 Centre for Health Economics, Faculty of Science, University of York, UK; 4 Liverpool Heart & Chest Hospital, UK

**Keywords:** Cardiac rehabilitation, prevention, percutaneous coronary intervention, observational study

## Abstract

**Aims:**

Despite the proven benefits of cardiac rehabilitation (CR), utilization rates remain below recommendation in the percutaneous coronary intervention cohort in most European countries. Although extensive research has been carried out on CR uptake, no previous study has investigated the factors that lead patients to attend the initial CR baseline assessment (CR engagement). This paper attempts to provide new insights into CR engagement in the growing percutaneous coronary intervention population.

**Methods and results:**

In total, we analysed data on 59,807 patients who underwent percutaneous coronary intervention during 2013 to 2016 (mean age 65 years; 25% female). Twenty factors were hypothesized to have a direct impact on CR engagement and they were grouped into four main categories; namely socio-demographic factors, cardiac risk factors, medical status and service-level factors. A binary logistic regression model was constructed to examine the association between CR engagement and tested factors. All but one of the proposed factors had a statistically significant impact on CR engagement. Results showed that CR engagement decreases by 1.2% per year of age (odds ratio 0.98) and is approximately 7% lower (odds ratio 0.93) in female patients, while patients are 4.4 times more likely to engage if they receive a confirmed joining date (odds ratio 4.4). The final model achieved 86.6% sensitivity and 49.0% specificity with an area under the receiver operating characteristic curve of 0.755.

**Conclusion:**

The present results highlight the important factors of the likelihood of CR engagement. This implies that future strategies should focus on factors that are associated with CR engagement.

## Introduction

Cardiac rehabilitation (CR), which is defined as a structured multidisciplinary intervention for cardiovascular risk assessment and management, advice on structured exercise training, psychosocial support and the appropriate prescription and adherence to cardio-protective drugs, is the most investigated form of secondary prevention interventions.^1^ CR has been established as the most clinically and cost-effective intervention in cardiovascular (CVD) disease management.^2^ CR improves clinical outcomes by modifying cardiac risk factors and is cost saving through a reduction in unplanned re-admissions for cardiac problems.^3^ Participation in a CR programme for patients hospitalized for an acute coronary event or revascularization is therefore recommended by European guidelines (class 1 level A).^4^ However, despite the proven benefits of CR it remains underutilized in many healthcare systems, with major inequities in access for certain patient groups such as the elderly and female patients.^5^ Furthermore, it has previously been observed that utilization rates are lower than expected in patients undergoing percutaneous coronary interventions (PCIs) in most European countries.^6^

Although extensive research has been carried out on CR uptake (e.g. proportion of eligible patients starting core CR), researchers have not investigated the factors that are associated with patients attending an initial CR baseline assessment (CR engagement), which informs the design of the tailored CR programme. Not all patients who attend the initial CR baseline assessment take part in the core CR programme, and not all patients that are eligible engage with CR at all. European guidelines continue to recommend CR initial assessment as a minimum standard and core component of CR.^7^

According to the British Association for Cardiovascular Prevention and Rehabilitation this baseline assessment could commence on a ward prior to discharge, or at an outpatient clinic or when the patient first attends the outpatient programme. It is only deemed complete when a formal assessment of lifestyle risk factors (smoking, diet, fitness and physical activity status), psychosocial health status, medical risk factors (blood pressure, lipids and glucose) and use of cardio-protective therapies has taken place.^2^

This paper aims to provide new insights into the factors that lead patients in the PCI population to attend their initial CR baseline assessment. We hypothesized that CR engagement is not a single patient decision but also is related to service level initiatives.

## Methods

This study investigates factors that will predict patient engagement with CR among PCI patients. A logistic regression model will be constructed to identify predictors of CR engagement among the selected population.

### Data source

The British Heart Foundation National Audit of Cardiac Rehabilitation (NACR) is operated in collaboration with NHS Digital to monitor the quality of and outcomes from cardiovascular secondary prevention and rehabilitation services in the UK. NACR has approval that is gained on an annual basis (under section 251 of the NHS Act 2006) to collect anonymized patient data for a range of clinical variables without the explicit consent from individual patients.^8^ Data are gathered by clinicians through validated questionnaires that are completed via a secure online system hosted by NHS Digital. The secure online data include details of patients’ demographic characteristics, clinical condition and lifestyle. NACR has shown to be representative of CR provision in the UK with 72% of all CR programmes entering data electronically using the NACR online system.^8^

To investigate the impact of social deprivation on CR uptake, the Index of Multiple Deprivation (IMD) 2010 was linked to the NACR dataset. The IMD is the official measure of relative deprivation for small areas (or neighbourhoods) in England.^9^ The IMD scores are based on eight distinct domains of deprivation with respect to income, employment, education, skills and training, health and disability, crime, barriers to housing and services, and living environment. These are combined, using appropriate weights, to generate an approximate overall deprivation score for each individual patient according to their small area of residence.^5^

### Design and inclusion criteria

**Figure 1.**
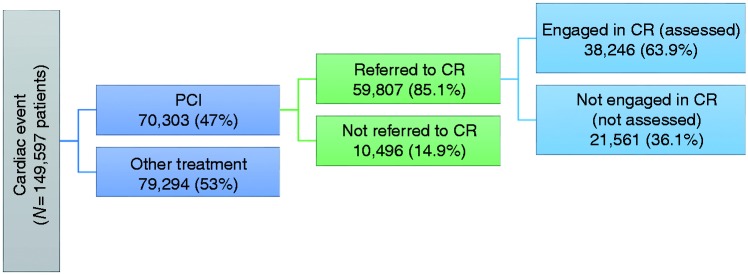
Study flow and sample size. CR: cardiac rehabilitation; PCI: percutaneous coronary intervention

This is a retrospective observational study using data retrieved from the NACR dataset for the period 1 April 2013 to 31 March 2016. Although NACR collects data for three countries (England, Northern Ireland and Wales), only patients in England were included in the study as the IMD is only available for English small areas. In addition, patients were included in the analyses if they had any type of PCI treatment during the study period and were referred to CR (Figure 1). Referral to a CR programme in England is usually conducted while the patient is still admitted or shortly after discharge for day case PCI patients.^5^

### Factors investigated

Twenty factors from the primary dataset were hypothesized to have a direct impact on patients’ decision to engage in CR based on the wider literature on CR uptake^10–15^ (Table 1). Predictor variables were either categorical or continuous depending on the method of data collection in NACR. The IMD score was grouped into five equal-sized quintile groups where the first quintile includes the most-deprived patients and the fifth quintile includes the least-deprived patients.

**Table 1.** Hypothesized predictors for CR engagement.

	Socio-demographic factors	Cardiac risk factors	Patient’s medical status	Service level factors
1	Age	High blood pressure	Total number of comorbidities	Referred to CR by
2	Sex	Diabetes	Previous cardiac event	Venue of source of referral to CR
3	Ethnicity	High blood cholesterol	Angina	Hospital length of stay
4	Marital status	Anxiety		Received confirmed joining date
5	Index of Multiple Deprivation	Depression		PCI type
6		Family history		Patient received early CR

CR: cardiac rehabilitation; PCI: percutaneous coronary intervention

### Data analysis

Descriptive statistics were calculated to compare differences in baseline characteristics between engaged and non-engaged patients. We used *t*-test for continuous variables and chi-square (χ^2^) tests for categorical variables with *p*-values < 0.05 considered to be statistically significant.

A binary logistic regression model was constructed to predict the probability of CR engagement and to examine the association among the research variables. We followed a backward selection process in which all variables were entered simultaneously in the model and variables with *p*-value >0.05 were removed. This process was repeated until all variables had *p* < 0.05. We also used forward selection techniques, beginning with a simple model including patients’ socio-demographic factors only, to which the other three blocks of predictors (Table 1) were then added in sequence to create three additional, increasingly more complex models. The four models were then tested against each other on the basis of log likelihood and variance explained (Pseudo-*R*^2^).

Since age and gender were reported in the literature as a major determinant of CR accessibility and outcomes,^8,16,17^ age and gender-specific interaction was tested by inserting a two-way age and gender interaction term in the model as a separate variable. To account for other interactions in the model between gender and any other tested variable, the analysis was repeated for males and females separately (stratified analysis).

The final model’s goodness-of-fit was evaluated using a Hosmer and Lemeshow test.^18^ To validate the model predictive power, a receiver operating characteristic (ROC) curve was plotted and model accuracy was measured by the area under the ROC curve (AUC).^19^

Under the assumption that missing values are missing at random, all variables with >5% missingness were handled by multiple imputation using 20 imputed datasets. The resulting estimates were pooled using Rubin’s rule. All analyses were performed using SPSS version 24.

## Results

The analysis sample included 59,807 patients. The baseline characteristics of both groups (engaged and not-engaged) are illustrated in Table 2.

**Table 2.** Baseline characteristics of both groups.

Factor	Engaged	Not-engaged	*p* value
*n*	38,246 (63.9%)	21,561 (36.1%)	<0.001
Mean age (SD)	64.16 (11.7)	65.36 (12.4)	<0.001
% Female	24.7%	25.6%	0.012
Ethnicity, White	85%	81%	<0.001
Marital status, single	23.3%	25.9%	<0.001
IMD* score (5)^a^	25.4%	19.3%	<0.001
% Comorbidities (+3)	30.1%	23.6%	<0.001
% Elective PCI procedure	35%	32.6%	<0.001
% Day case procedure	15.9%	18.4%	<0.001

a Ratio of least deprived patients in the cohort.

*IMD: Index of Multiple Deprivation; PCI: percutaneous coronary intervention

Table 3 compares the summary statistics for the four models created as explained in the methods section.

**Table 3.** Summary statistics for the four models created by forward stepwise regression.

Model	–2 Log likelihood ratio	Pseudo-*R*_2_	Correctly classified cases
Model 1^a^	77226.16	0.02	63.9%
Model 2^b^	73698.13	0.03	64%
Model 3^c^	72608.09	0.05	64.8%
Model 4^d^ (final)	63847.12	0.25	73.1%

a Socio-demographic factors only.

b Model 1 plus risk factors.

c Model 2 plus patient’s medical status.

d Model 3 plus service level factors.

The final model was statistically significant, χ^2^ (32) = 11,928.8, *p* < 0.0005. The model explained 25% (Nagelkerke *R*^2^) of the variance in CR engagement and correctly classified 73.1% of cases. Sensitivity was 86.6%, specificity was 49%, positive predictive value was 75.1% and negative predictive value was 67.3%. The ROC curve test indicates that the final model has a good predictive ability with AUC of 0.755 (SE = 0.002, 95% confidence interval (CI), 0.751 to 0.759). To assess the model for influential cases, Cook’s distance test and leverage values were computed. There were no unusually high values in both tests (all < 1). Hosmer and Lemeshow test in the final model is not statistically significant (*p* = 0.349), indicating that the model is not a poor fit. Of the 20 predictors tested, only hypertension was found to be not statistically significant (Table 4). Splitting the data into male and female groups to account for gender related interaction with other variables did not reveal any significant change in the reported results.

**Table 4.** Pooled estimates of the logistic regression model predicting likelihood of CR engagement.

Factor^a^	Categories	*p* value	OR	95% CI for OR	
				Lower	Upper
Age	In years	0.000	0.988	0.987	0.990
Sex (male)	Female	0.002	0.929	0.885	0.974
Ethnicity (White)	Ethnicity Black	0.222	1.239	0.878	1.749
	Ethnicity South Asian	0.001	0.866	0.792	0.946
	Ethnicity Other	0.000	0.757	0.712	0.804
Marital status (single)	In partnership	0.000	1.223	1.144	1.307
	Previously partnered	0.000	1.250	1.153	1.355
IMD rank (1 most derived)	IMD rank (2)	0.480	1.029	0.951	1.113
	IMD rank (3)	0.000	1.190	1.101	1.288
	IMD rank (4)	0.000	1.240	1.155	1.331
	IMD rank (5)	0.000	1.464	1.363	1.572
Cardiac risk factors (no)	Hypertension	0.407	0.977	0.923	1.033
	Diabetes	0.000	0.877	0.822	0.935
	Depression	0.000	1.561	1.374	1.774
	Hypercholesterolaemia	0.000	0.787	0.743	0.834
	Family history	0.005	1.093	1.027	1.162
	Angina	0.000	1.225	1.144	1.312
	Anxiety	0.000	1.435	1.257	1.639
Number of comorbidities (0)	Comorbidity < 3	0.000	1.589	1.477	1.710
	Comorbidity > 3	0.000	1.802	1.586	2.048
History of previous cardiac event (no)	Previous event	0.000	0.749	0.715	0.786
Patient refereed by (consultant)	Cardiac nurse	0.000	0.902	0.854	0.953
	GP	0.467	1.791	0.348	9.204
	Primary care nurse	0.056	1.391	0.992	1.953
	Other	0.085	1.097	0.987	1.219
Venue of source of referral (NHS Trust)	General Practice	0.000	9.302^b^	7.803	11.091
	BMI/private hospital	0.035	0.810	0.667	0.985
Hospital length of stay (overnight stay)	Day case	0.000	0.736	0.691	0.784
Received confirmed joining date (no)	Yes	0.000	4.443	4.239	4.656
PCI type (primary)	MI	0.000	1.111	1.060	1.165
	Elective	0.000	1.211	1.146	1.278
Patient received early CR (no)	Yes	0.000	0.533	0.509	0.558
Constant	–	0.000	5.602	3.830	8.194

a Predictor with base category in brackets, ^b^the effect inflated by small sample size.

CR: cardiac rehabilitation; OR: odds ratio; CI: confidence interval; IMD: Index of Multiple Deprivation; GP: General Practitioner; NHS: National Health Service; BMI: BMI Healthcare; PCI: percutaneous coronary intervention; MI: myocardial infarction

## Discussion

This is the first study to investigate the determinants of CR engagement in patients following PCI treatment. In this retrospective secondary analysis, it was found that the probability of CR engagement decreases by 1.2% (odds ratio (OR) 0.98, 95% CI 0.987 to 0.990) per additional year of patient age, and is approximately 7.1% lower (OR 0.929, 95% CI 0.885 to 0.974) for female patients compared with male patients. These novel results, obtained from routine clinical data, support the findings of earlier systematic reviews and meta-analyses which indicate that existing CR programmes are more attractive to middle-aged male patients, thus perhaps being less attractive to the elderly or female patients.^7,20–22^

The recent European guidelines on CVD prevention have emphasized that minority ethnic groups such as South Asians have a higher risk of CVD but are less represented in CR programmes.^15,23^ Our results support this and suggest that South Asians are less likely to engage in CR compared with the majority ethnic White patient population (OR 0.866), thereby identifying a potential mechanism that leads to differential uptake of CR programmes. Also, CR engagement was significantly correlated with the index of social deprivation as measured by IMD where CR engagement increased from the most deprived to the least deprived patients (except for the first two most deprived deciles). Current European and international guidelines have called for equal access for all myocardial infarction (MI) patients, including those from minority ethnic groups and socially deprived groups, and our results question the extent to which this has been achieved.^10,24,25^ In addition, single patients are less likely to be engaged in CR compared with partnered or previously partnered patients (22% and 25% respectively). This may be because couples facilitate attendance by providing social support, transportation to CR centres or communication with health professionals.^11^ However, note that previously partnered patients are the most engaged CR group.

The current study found that cardiac risk factors play a major role in CR engagement. Diabetes (OR 0.88), hypercholesterolaemia (OR 0.79) and history of previous cardiac event (OR 0.749) are associated with reduced CR engagement while hypertension was not found to be a significant predictor of CR engagement (*p* = 0.404). Other risk factors such as angina (OR 1.22), anxiety (OR 1.43), depression (OR 1.56) and family history of cardiac disease (OR 1.09) were found to increase the likelihood of patients’ engagement in CR. One unanticipated finding was that the number of comorbidities was not found to be in itself a barrier to CR engagement. This finding contradicts a retrospective analysis conducted in The Netherlands^12^ and in another, Canadian, qualitative study,^26^ although these studies were investigating uptake to core CR not CR engagement, that is, the initial baseline assessment that may take place before or at the very beginning of core CR sessions.

If patients had a life-saving PCI (primary PCI) they were less likely to engage in CR compared with MI/PCI and elective PCI (OR = 1.21 and OR = 1.11). Having PCI as a day case procedure also reduced the likelihood of CR engagement by 27%. This result may be explained by the fact that a day case procedure reduces the time window to identify and recruit patients to CR thus requiring programmes to be more innovative in contacting patients.^5^ Another finding that was contrary to expectations is that patients who took part in early phase 1 CR sessions (either inpatient or home-based programmes) were less likely to start the core CR programme (OR = 0.533).

One of the most telling finding to emerge from the analysis is that patients who were given a firm date to attend the initial CR assessment were over four times more likely to engage in CR (OR 4.443). Also, patients who have been referred from a general practice were more than nine times more likely to attend the assessment session compared with patients referred from a hospital setting (OR 9.30). The primary route of referral in our sample was through a cardiac nurse (74.7% of patients), and these patients were significantly less likely to engage in CR compared with patients referred by consultant, general practitioner or primary care nurse (OR 0.902). It is difficult to explain this result; however, the strength of healthcare professional endorsement for CR is known to play a significant role in CR uptake.^13^

The analysis of CR engagement undertaken here has extended our understanding of the determinants of low CR utilization rates in England. Although age and gender are significant determinants of CR engagement, which is also true for CR uptake, Table 3 illustrates how service level factors play a major role in CR engagement. These findings highlight that service level initiatives, such as providing a firm date to attend the initial CR baseline assessment, play an important part in promoting initial CR engagement. Further research should be undertaken to investigate the differences and determinants between those patients who start CR and those who drop out.

### Study limitations

Since the NACR dataset is set up to evaluate final outcomes but not CR engagement, it is possible that some other relevant factors influencing CR engagement have been missed. Also, while we evaluated the type of PCI as a determinant of uptake, it is likely that these correlate with unobserved clinical factors, so that our estimate of the effect of PCI type may be subject to confounding.

## Conclusion

This is the first study on CR engagement from a nationally representative cohort of patients. This paper provides new insights into the factors that lead patients to attend their CR initial baseline assessment (CR engagement) in the growing PCI population. The most obvious finding to emerge from this study is that CR engagement is not a single patient decision but also is related to service level factors, over which healthcare systems have more direct control. The findings should make an important contribution to our understanding of the relatively low CR utilization rates in this cohort despite the known benefits of CR.

## Author contribution

AQ, PD, NG and JM contributed to the conception and design of the work. AQ performed the statistical analysis. PD handled funding and supervision of the work. AQ and PD acquired the data and drafted the manuscript. AQ, PD, NG and JM critically revised the manuscript. All gave final approval and agree to be accountable for all aspects of work ensuring integrity and accuracy.
